# Lambda restricted light chain as pathogenic cause of POEMS syndrome: a review of the hypothesis

**DOI:** 10.3389/fimmu.2026.1865997

**Published:** 2026-07-06

**Authors:** Rebecca Troutman, Angela Wagner, Elizabeth Delery

**Affiliations:** 1 Wood College of Osteopathic Medicine, Marian University, Indianapolis, IN, United States; 2 Indiana Biosciences Research Institute, Indianapolis, IN, United States; 3 Department of Biochemistry, Molecular Biology and Pharmacology, Indiana University School of Medicine, Indianapolis, IN, United States

**Keywords:** antibody, LAMBDA, light-chain, plasma cell, POEMS

## Abstract

POEMS syndrome is a rare and chronic paraneoplastic syndrome characterized by polyneuropathy, organomegaly, endocrinopathy, monoclonal protein, and skin changes. The pathophysiology of this disease is not well understood, but current research focuses on the increase in VEGF and the highly restrictive nature of λ light chains produced by monoclonal plasma cells. Recently, studies have looked deeper into the possibility of these λ light chains being a major factor in onset and progression of this disease focusing on molecular mimicry and links to viral infections. This mini review will summarize current literature on POEMS syndrome as well as hypothesize and analyze the role of the λ light chain in the pathophysiology of the disease.

## 1 Introduction

POEMS syndrome (Polyneuropathy, Organomegaly, Endocrinopathy, Monoclonal Protein, and Skin changes) is a rare paraneoplastic syndrome caused by an underlying plasma cell disorder that falls into the category of Monoclonal Gammopathy of Clinical Significance (MGCS) (1). The diagnosis of POEMS syndrome requires two mandatory, at least one major, and at least one minor criteria, which can be found in **Table 1**. Many other symptoms may be associated with this disease, as it affects almost all organ systems, but are not required for diagnosis (1–4).

**Table 1:** Diagnostic criteria of POEMS syndrome and other associated symptoms.

Table 1	
Mandatory Criteria	• Polyneuropathy • Monoclonal B-cell proliferation
Major Criteria(at least one)	• Sclerotic bone lesions • Castleman disease • Elevated VEGF
Minor Criteria(at least one)	• Organomegaly • Volume overload • Endocrinopathy
Additional Associations	• Pulmonary symptoms • Central nervous system (CNS) involvement • Arterial vascular disease • Renal disease

The pathogenesis of POEMS syndrome remains poorly understood, in large part due to its rarity in the population and the complexity of the disease itself (1, 2). POEMS syndrome shares many of its presenting characteristics with other conditions, such as Chronic Inflammatory Demyelinating Polyneuropathy (CIDP) and Multiple Myeloma (MM), which adds to the complexity of understanding pathogenesis due to a delay in accurate diagnosis (4).

Recent research has emphasized the increase in serum vascular endothelial growth factor (VEGF), along with other pro-inflammatory cytokines, as being a major causative factor in the characteristic symptoms of POEMS syndrome (2). It has been hypothesized that VEGF is responsible for the symptoms of peripheral neuropathy, organomegaly, and volume overload that are seen with POEMS syndrome (3). The source of the increased VEGF has been thought to be from monoclonal bone marrow plasma cells (5), however newer studies have produced controversial results finding that the amount of VEGF mRNA present in plasma cells of POEMS syndrome is no higher than that found in other plasma cell disorders (6, 7).

In addition to germline restrictions, common mutations are also seen across POEMS patients within these germlines. While it is unclear what pathogenic role these restrictions play, recent studies have found they may play a role in the increase of serum VEGF levels and provide a link between viral infections and the onset of POEMS syndrome (8, 10). In addition to their possible role in the pathogenesis of this disease, these features are unique to POEMS syndrome and can be used as an important factor in diagnosis (7, 10).

## 2 Immunoglobulin restriction in POEMS

Immunoglobulin sequencing has long been used to determine plasma cell features of different plasma cell disorders. Sequencing of *IGLV* has previously been done for multiple myeloma, monoclonal gammopathy of undetermined significance (MGUS), and amyloid light chain (AL) amyloidosis (6, 8). This sequencing can provide some insight into the characteristics of plasma cells associated with these diseases and provide information that can help with differentiation and diagnosis of plasma cell disorders. More recently, research has been conducted on the specific characteristics of plasma cells that are associated with POEMS syndrome.

Unlike other plasma cell disorders, monoclonal plasma cells associated with POEMS syndrome are highly restricted. A unique feature of POEMS syndrome is that plasma cells are lrestricted. Interestingly, the immunoglobulin light chain (IGL) variable regions (V) are further restricted to the Vl1 subfamily and limited to two main germlines, IGLV1–40 and IGLV1-44 (7–9). Multiple researchers have hypothesized that due to the highly restrictive nature of the λ light chains, they play an important role in the pathogenesis of POEMS syndrome, although that role remains unclear (8, 10). While restricted germline usage does not guarantee pathogenicity of these λ light chains, these restrictions are still a useful tool in the diagnosis of POEMS syndrome because IgAλ or IgGλ restricted plasma cells are required for diagnosis (2).

### 2.1 Heavy chain

Research regarding POEMS antibodies has focused on light chain restrictions. Few studies have mentioned heavy chain germlines seen in POEMS syndrome due to no clear patterns or restrictions seen with the heavy chains. Plasma cell heavy chains in POEMS syndrome are not restricted to one specific heavy chain class, although heavy chain type α (IgA) or type γ (IgG) is required for POEMS diagnosis (2, 3). When sequencing λ light chain genes, one research group also sequenced the immunoglobulin heavy chain variable region (*IGHV)* genes from five different patients. All five genes were found to be from different *IGHV* germlines, with no clear pattern between them (10).

### 2.2 Light chain

Numerous studies have found restrictive usage of immunoglobulin λ light chain genes in POEMS syndrome (7–9). These genes are typically restricted to *IGLV1–40* or *IGLV1–44* germlines, with some studies also reporting a lower prevalence of *IGLV1-36* (10, 11). One of the initial studies that found the restriction of λ light chains to *IGLV1–40* and *IGLV1–44* found that this restriction has no more than a 1% exception across POEMS patients (9). The highly restrictive nature of these germlines suggests that they play an important role in the pathogenesis of POEMS syndrome.

There is further restriction within these germlines, seen through specific mutations that are conserved across POEMS patients. Researchers found that all *IGLV1–44* sequences contained mutations at amino acid position 38, with a threonine being replaced by a hydrophobic residue in 89% of cases, most commonly proline, followed by alanine. They also found that histidine 40 was replaced by asparagine in 83% of patients and glutamine in 17% of patients that contained the *IGLV1–44* sequence. Interestingly, these mutations led to an amino acid consensus sequence of PVNWYQ in framework region 2 (FR2) of the *IGLV1-36*, *IGLV1-40*, and *IGLV1–44* sequences (10). This specific consensus sequence may play a role in the pathogenesis of this disease, as discussed in later sections. Other studies have found these same amino acid changes from germline (7, 8), confirming their specificity to POEMS syndrome.

Adding to the specificity of POEMS immunoglobulins, each complementarity-determining region 3 (CDR3) of immunoglobulin light chains of POEMS patients were composed of 11 amino acids with identical isoelectric points and similar molecular weights (8). This adds to the probability that λ restricted light chains are pathogenic in POEMS syndrome as there are so many commonalities seen across light chains in different POEMS patients.

One study was able to link different light chain variable region genes with differences in clinical features in patients with POEMS syndrome. This study found that individuals with *IGLV1–40* were typically younger at age of disease onset than those with *IGLV1-44*, and that those with *IGLV1–40* also had more severe neuropathy, hypertrichosis, and papilledema (12). While there were no other significant differences observed between these two groups, it is still worth noting the possible link of IGLV germline types with clinical features of the disease.

Not only has germline restriction been seen in the variable (V) region of immunoglobulin genes, but there has also been reported to be a junction (J) region restriction to IGLJ3*02 germline. This J region restriction was found in 100% of POEMS patients, regardless of their germline V region across several studies (7, 8, 10). This restriction further differentiates immunoglobulins found in POEMS patients from immunoglobulins found in other plasma cell disorders, pointing to its potential role in the pathogenesis of POEMS syndrome.

## 3 Role in pathogenesis

The disease mechanism of POEMS syndrome remains unknown, but many researchers believe that the increase in serum VEGF is responsible for many of the disease’s characteristics. Multiple studies have found that VEGF levels correlate best with disease activity and have found that increased VEGF may be responsible for many symptoms of POEMS syndrome (2–4). VEGF is known to play a role in angiogenesis and endothelial cell survival, enhancing vascular permeability and inducing vascular leakage. This offers an explanation to the manifestations of volume overload as well as neuropathy that may be due to endoneurial edema (1, 13).

The origin of this VEGF overproduction is a point of controversy as some studies say the increase comes from plasma cells (5) while others found no increase in VEGF mRNA within plasma cells of POEMS patients compared to plasma cells of patients with other plasma cells disorders (6, 7). More recently, studies have pointed to the possibility that λ light chains are responsible for the increase in VEGF through interactions with VEGF-related proteins (8, 10).

### 3.1 Heat shock protein 90 alpha homology

When studying the amino acid sequences and common mutations found in λ light chains, researchers found that the PVNWYQ amino acid consensus sequence had 100% homology with the heat shock protein (HSP) 90α chaperone protein (10). HSP90α is known to play a role in the regulation of angiogenesis and in the secretion of VEGF via hypoxia-inducible factor-1 (HIF-1) activation (13, 14). Due to this fact, they hypothesized that the λ light chain in POEMS syndrome causes the increased secretion of VEGF through molecular mimicry with HSP90α. More research is needed focusing on this homology as a possible cause of the increased VEGF, as short sequence amino acid homologies are a relatively common occurrence, and this HSP90α mimicry is still speculative. However, this specific amino acid sequence is highly specific to POEMS syndrome, and its homology to HSP90α offers potential insight into the pathomechanisms at work in this disease.

In addition to HIF-1 being responsible for VEGF secretion, it also plays a role in regulating the immune function of various immune cell types, such as dendritic cells, macrophages, and effector T cells (15). Molecular mimicry with HSP90α could also explain additional symptoms that are characteristic of POEMS syndrome such as the increase in pro-inflammatory cytokines.

HSP90α also has immune regulatory functions of its own. This chaperone protein is known to facilitate the folding of T cell and natural killer (NK) cell receptors, which promotes stimulation of these cells. Extracellular HSP90α can also serve as a damage-associated molecular pattern (DAMP). DAMPs drive an innate immune response resulting in cytokine secretion, such as IL1-β, TNF-α, IL-6, and IL-12 (15, 16), all of which are elevated in POEMS syndrome (3).

According to a review conducted by Binder, an increase in HSPs in the extracellular environment can trigger autoimmunity (16). This is most likely due to the pro-inflammatory conditions that HSPs, specifically HSP90, elicit. In addition to eliciting pro-inflammatory conditions, HSP90α is capable of chaperoning proteins that are cross-presented by antigen presenting cells (APCs). (16). This is due to the presence of cluster of differentiation 91 (CD91) on APC cell surfaces, which acts as a signaling receptor for HSPs. Autoimmunity can then easily be triggered by HSPs because the majority of proteins that HSPs chaperone are self-peptides (16).The pathogenicity of POEMS λ light chains remains unclear; However, multiple researchers hypothesize that the λ light chains are responsible for the increase in VEGF, which is thought to be a major driving factor in the characteristic POEMS symptoms (8, 10). While short sequence homologies are a relatively common occurrence, molecular mimicry of these λ light chains to HSP90α provides possible insight into how POEMS plasma cells may be responsible for the increase in VEGF along with the increase of numerous pro-inflammatory cytokines that are characteristic of POEMS syndrome.

### 3.2 Autoreactivity to type IV collagen

As mentioned previously, POEMS λ light chains may trigger autoimmunity from molecular mimicry to HSP90α. However, they may also trigger autoimmunity by being autoreactive to type IV collagen. Type IV collagen is an important structural component of the basement membrane. Different isoforms of type IV collagen are found in different locations in the body (17).

Foster et al. conducted a study to look for human antibodies that are reactive with α3(IV)NC1 collagen, a type IV collagen isoform found in the kidney, lung, cochlea, and eye (17). They found a total of six human antibodies that were reactive with this basement membrane collagen, one of them containing a λ light chain that uses *IGLV1–40* and IGLJ3*02 genes (18), which in combination are highly specific to POEMS syndrome. While this autoantibody uses a specific heavy chain gene, unlike those in POEMS, the specificity of the λ light chain genes to POEMS syndrome is something to be considered.

In addition to using *IGLV1–40* and IGLJ3*02 as specific λ light chain genes, each of the six autoantibodies discovered contained long to exceptionally long heavy complementarity determining region 3 (HCDR3) (18). It is uncommon for antibodies to contain long to exceptionally long HCDR3, making it especially uncommon for all known antibodies that are autoreactive to α3(IV)NC1 collagen to share this feature. The researchers that conducted this study hypothesized that the length of HCDR3 is an important factor in determining these antibodies autoreactivity, possibly more so than the immunoglobulin heavy chain (IGH) genes used (18).

While the pathophysiology of λ light chains in POEMS syndrome is not well understood, there is a possibility that these light chains are autoreactive to α3(IV)NC1 collagen. Autoreactivity to α3(IV)NC1 collagen would account for the papilloedema and renal disease seen in some POEMS patients. Autoreactivity to other isoforms of type IV collagen would also account for skin changes, endocrinopathy, and organomegaly that is commonly seen in POEMS patients (2, 17). An immunoglobulin λ light chain that uses *IGLV1–40* and IGLJ3*02 germlines is highly specific to POEMS syndrome, and even though POEMS plasma cells do not use specific heavy chain genes, not enough research has been conducted over POEMS heavy chains to determine if the HCDR3 follows the pattern of having long to exceptionally long amino acid sequences. Further research into POEMS heavy chains and the HCDR3 may provide insight into the possible autoreactivity of POEMS antibodies.

## 4 Link to viral infections

Some of the earliest studies over POEMS syndrome mention a possible link between the disease and viral infections (19). Researchers have tried to pinpoint what viral infection may be linked with this disease with controversial results. Some studies found a link between Human Herpesvirus 8 (HHV8) infection with POEMS syndrome-associated multicentric Castleman’s disease (20) while another study argues that while HHV8 is a strong candidate for the pathogenesis of POEMS syndrome, the infection rate of POEMS syndrome patients and the general population is not different enough to conclude that HHV8 infection is related to the onset of POEMS syndrome (21). In addition to this, links between HHV8 and POEMS have only been established in patients who have also been diagnosed with multicentric Castleman’s disease (20). While multicentric Castleman’s disease is one of the major criteria for diagnosis with POEMS syndrome, it is not required for diagnosis (1-3). While no link has been established to a specific viral infection, some studies still point to some viral infection being a possible trigger to POEMS syndrome (20), and additional research into other viral infections could lead to a deeper understanding of the possible link between viral infections and the onset of POEMS syndrome.

Multiple studies found a higher ratio of replaced/silent (R/S) mutations in CDRs than in framework regions (FWRs) of *IGLV1–40* and *IGLV1–44* germlines (8, 12). The higher ratio of R/S in CDRs than FWRs strongly suggests that the clonal plasma cells have undergone antigen selection, indicating a response to an infection, and more specifically that this selection occurred before any sort of cancer transformation or disease onset (9).

Other studies hypothesize that there may be a specific viral infection that triggers the onset of POEMS syndrome, which would explain the lack of diversity among the immunoglobulin light chains that are so unique to POEMS syndrome (7, 20). While more research is needed to specify a specific viral culprit, this possible link to viral infections provides further evidence to suggest that the λ light chains are a major causative agent in the characteristic symptoms of POEMS syndrome.

## 5 Discussion

POEMS syndrome is a rare plasma cell disorder with unknown pathophysiology. One of the major criteria for diagnosis with POEMS syndrome is an increase in serum levels of VEGF. This increase in VEGF, along with increases in several pro-inflammatory cytokines, is thought to be the cause of many of the characteristic symptoms of POEMS syndrome, including polyneuropathy, which is required for diagnosis (2–4). The source of the increase in VEGF has caused some controversy with different studies obtaining contradictory results. Some researchers believe the increase in VEGF is caused by direct production from monoclonal plasma cells while others believe the increase could be indirectly caused by the immunoglobulins produced by these plasma cells (5–7).

Recent studies have placed emphasis on the possible pathogenic role of λ restricted light chains due to their highly restrictive nature to two specific germlines, *IGLV1–40* and *IGLV1-44* (7–9). Further supporting the pathogenic role of λ light chains is the link of POEMS syndrome to viral infections. Given that multiple studies have found that clonal plasma cells undergo antigen selection prior to disease onset or progression (7, 9), some researchers believe that a viral infection is the initiating factor in the production of clonal plasma cells in POEMS syndrome, and that onset and progression of the disease does not begin until plasma cells have selected these germlines for use in creating antibodies. The highly restrictive nature of λ light chains suggests a single viral infection may be to blame.

Further supporting this hypothesis, other studies have found that the λ light chain may be responsible for the increased production of VEGF along with increased production of pro-inflammatory cytokines and further immune stimulation (13–16). This increase in VEGF, along with possible autoreactivity to type IV collagen (18), would explain the characteristic symptoms of POEMS syndrome.

## 6 Future research

Future research looking into the possible autoreactivity of λ light chains to type IV collagen could provide insight into the pathogenicity of these λ restricted light chains. Researching what these specific immunoglobulins are reactive to could provide insight into the causes of some of the characteristic symptoms associated with POEMS syndrome. It could also further solidify the link between POEMS syndrome and viral infections if these antibodies are found to be protective against known viruses.

Studies could also be conducted to determine if λ light chains are capable of interacting with VEGF-related proteins, such as HIF-1, in place of HSP90α. This could determine the cause of the increase of VEGF commonly seen in POEMS syndrome and explain some of the other symptoms seen with this disease. While there are numerous studies supporting λ light chains as a major causative agent in POEMS syndrome, more research is needed to definitively determine the pathogenic cause of POEMS syndrome.
